# Online Assessment of Individual Differences in Memory and Cognition: Risk and Resilience

**DOI:** 10.1002/alz70857_099510

**Published:** 2025-12-24

**Authors:** Brian Levine, Ryan Yeung, Nicholas Diamond, Stephanie Simpson, Stuart Fogel, Moriah Sokolowski, Carina L Fan, Daniel Baena

**Affiliations:** ^1^ Rotman Research Institute, Baycrest Academy for Research and Education, Toronto, ON, Canada; ^2^ Baycrest, Toronto, ON, Canada; ^3^ Rotman Rsearch Institute at Baycrest, Toronto, ON, Canada; ^4^ Rotman Research Institute at Baycrest, Toronto, ON, Canada; ^5^ University of Ottawa, Ottawa, ON, Canada; ^6^ Toronto Metropolitan University, Toronto, ON, Canada

## Abstract

Impaired episodic memory is a hallmark sign of medial temporal lobe damage from Alzheimer's disease. There is systematic variability across individuals in episodic memory capacity, particularly for autobiographical memory—or memory for events from one's life—the putative real‐life correlate of laboratory tests of episodic memory, and one that is heavily dependent on visual imagery. Individuals with congenitally low episodic memory and visual imagery may have advantages on conceptual tasks that rely on abstraction of patterns across episodes. We address this hypothesis across three independent studies from our research on episodic memory, all of which included a set of tests from the Creyos battery assessing visual memory (Paired Associates), spatial working memory (Mental Rotations), deductive reasoning (Odd One Out), and syntactic/grammatical reasoning (Grammatical Reasoning). In 2357 participants tested online, trait episodic memory and visual imagery abilities were negatively correlated with computational elements of STEM professions. In 949 participants exposed to trauma, visual imagery was negatively correlated with clinical symptoms following exposure to psychological trauma. In 93 participants in a study on sleep and memory, sleep (vs. wake) and sleep spindles as measured with full polysomnography related to improvements in sequence memory for real‐life events. Across all three studies, the observed findings were related to performance on the Creyos Grammatical Reasoning test (but not other Creyos measures). These findings suggest that abstract reasoning opposes episodic memory at the level of individual differences. In turn, they suggest that reasoning abilities can counteract low memory and potentially confer resilience against medial temporal lobe dysfunction.

